# Evaluation of a new goal-directed training curriculum for point-of-care ultrasound in the emergency department: impact on physician self-confidence and ultrasound skills

**DOI:** 10.1007/s00068-019-01126-0

**Published:** 2019-04-08

**Authors:** Di Shi, Jihai Liu, Jun Xu, Huadong Zhu, Xuezhong Yu

**Affiliations:** Department of Emergency Medicine, Peking Union Medical College Hospital, Peking Union Medical College, Chinese Academy of Medical Sciences, No. 1 Shuai Fu Yuan, Dong Cheng District, Beijing, 100730 China

**Keywords:** Emergency medicine, Ultrasound, Education

## Abstract

**Purpose:**

Developing countries need effective and efficient training curriculum for the point-of-care ultrasound (POCUS) in the emergency department (ED). We have developed a new goal-directed training curriculum focusing on critical POCUS procedures used in ED.

**Methods:**

To evaluate the impact of the new POCUS curriculum on ED physicians’ performance/self-confidence, we carried out a quasi-experimental trial at ED training center of Peking Union Medical College Hospital, in which we trained two groups of physicians using either traditional curriculum or the new goal-directed curriculum. We measured the confidence in performing Focused Assessment with Sonography in Trauma, thoracic, vascular, ultrasound-guided puncture, echocardiography and undifferentiated shock diagnostic ultrasound at baseline, training completion and 1 month after training. We also measured the performance skills at the time of training completion. The relative value of the new curriculum was evaluated by differences in the confidence and performance measurements, with control for baseline measurements and confounding characteristics in univariate analyses and multivariate linear regression models.

**Results:**

After training, both groups of trainees reported significantly increased self-confidence in performing POCUS as compared with baseline. Trainees with the new curriculum had statistically significant higher self-confidence increase and performance scores (*p < *0.05), e.g., increase in confidence for diagnosis of undifferentiated shock at training completion = 3.14 vs. 1.85, 1 month after training = 2.22 vs. 1.56, for new and traditional curriculum, respectively. The new curriculum also resulted in a higher number of shock POCUS done within 1 month after training: 1.73 vs. 0, and higher overall performance scores: 165 vs. 113. The findings were robust when controlling for imbalanced baseline characteristics in multivariate regression models.

**Conclusion:**

We conclude that a goal-directed, intensive but brief ED POCUS curriculum significantly increases trainee self-confidence, performance, and promote trainees to perform more procedures.

## Background

Many clinical settings have adopted the point-of-care ultrasound (POCUS) in practices [1–5]. Recognizing the value of the POCUS and the need for adequate training, various clinical settings have developed POCUS training curriculums [6–11]. The unique characteristics of emergency clinical workflow require dedicated training for POCUS in the emergency department (ED). ED physicians need to perform the image acquisition on the spot and extract information in a timely fashion to aid the patient diagnosis and treatment. On the other hand, the application of POCUS in ED can be intensely focused on the specific use cases and often driven by common goals of emergency medicine (EM) patient management.

Although various EM POCUS training guidelines have been developed [12–14], POCUS training curriculums in ED remained quite varying between institutions, as recognized by the International Federation for Emergency Medicine in 2015 [6, 15]. Typical curriculums usually take several days including hours of didactic training followed by practices on proctored scans. The lengthy sessions of studying the course materials, interactive online learning, modular learning, and hands-on experiences are to ensure the trainee will acquire the knowledge and skills to efficiently utilizing POCUS. However, time-consuming sessions are expensive to institute, especially for developing countries with a considerable gap in physician POCUS expertise and limited resources. Curriculums that delivers both efficiency and quality are in great need to enable trainees in achieving a solid understanding of the anatomic structure, obtaining high-quality images in standard views, recognizing pathological findings and supporting clinical decisions.

With the aid of new technology of 3-D ultrasound image recording, we have developed a new, brief, goal-directed curriculum for POCUS training in ED. We expect the goal-directed curriculum, requiring the same amount of training time, will outperform the previous standard curriculum by improving trainee self-confidence and increasing performance carrying out POCUS. This study aimed first to introduce the new brief POCUS curriculum and then to evaluate the impact of the new curriculum on trainee performance/self-confidence in relative to the standard curriculum.

## Methods

### Study design and participants

This study was a quasi-experimental trial carried out to investigate the impact of two different POCUS curriculums on ED physicians’ performance/self-confidence. We conducted the trial at the ED training center of Peking Union Medical College Hospital (PUMCH). We recruited participants enrolled in PUMCH emergency POCUS training course from 28 December 2015 to 17 June 2017. Trainees enrolled before 31 December 2016 underwent training using the traditional curriculum, and those enrolled after received training using the new curriculum. As the enrollment to the PUMCH bedside ultrasound training course was open to all interested emergency physicians nationwide, there was no requirement for prior ultrasound education or hands-on experiences, and no specific control for trainees’ allocation to either the traditional or the new goal-directed curriculum.

We approached the two consecutive cohorts of trainees for consent and included only consent trainees in the study. We further excluded trainees who failed to finish the course or/and did not respond to follow-up. The institutional board of PUMCH reviewed and approved the study, considering this study presented no more than minimal risk to subjects.

### Study interventions

For both standard and new curriculums, trainees participated in a two and half day training course in which they formed study groups of about four to seven members. One or two teaching physicians were responsible to coach individual study group in hands-on sessions.

### Traditional curriculum

Subjects enrolled in the training program before 31 December 2016 received training using a traditional curriculum. The curriculum started with an introductory lecture on the fundamental ultrasound knowledge, which covered physics and instrumentation of emergency ultrasound, followed by lectures on how to obtain standard ultrasound views without emphasis on relevant ultrasound anatomy, or normal/pathological images interpretation. After the educational lectures, the program provided hands-on practices on simulated manikins for trainees to obtain standard views with the guidance of instructors. The curriculum completed with the introduction of Rapid Ultrasound for Shock and Hypotension (RUSH) protocol and a lecture for using POCUS to aid the diagnosis of undifferentiated shock.

### New goal-directed curriculum

Subjects enrolled in 2017 underwent training using an improved, goal-directed curriculum. We identified the five core applications that ED physicians commonly perform to assess patients in their anatomical structures and underlying pathophysiological changes: Focused Assessment with Sonography in Trauma (FAST), thoracic, vascular, ultrasound-guided puncture (UGP) and echocardiography. Details of the five ultrasound applications are included in Appendix 1. The learning objectives of the training were to facilitate trainees to correctly carry out the above five ultrasound applications and obtain accurate assessment through a limited number of standard views. Also, we retained our emphasis on the POCUS procedure for undifferentiated shock diagnosis and introduced the “Tamponade/Tension, pneumothorax, Heart, Inferior vena cava, Respiratory system, Deep venous thrombosis/Aorta Dissection” (THIRD) procedure in the new curriculum [16].

Before formal courses, we provided trainees with pre-course learning materials to familiar with the necessary knowledge of ultrasound and the consensus of evaluation for undifferentiated shock patients. The pre-course materials contained reading literature and web-based video sessions. The ED of PUMCH has published details about the pre-course learning in May 2017 [16].

During the formal course, we improved the first introductory lecture for basic ultrasound knowledge by integrating hands-on practice with coaching, which included ultrasound probe holding, probe placement/movements, understanding of three ultrasound plains of tridimensional organ, explanation on four sides of the view, and summary on six steps of obtaining a high-quality view.

After the introductory lecture on basic ultrasound knowledge, we also changed the sequence of subsequent training sections with the improvement of contents and technologies. Subjects had the first lecture on the introduction of anatomy with 3D video records and photos of dissecting real organ. The second lecture provided the tips to gain a standard view in details and compared 3D images with normal ultrasound views. Trainees practiced drawing ultrasound views on simulated manikins after this session. Each trainee was assigned to a group for hands-on practice instructed by group instructor, the ratio of instructor to trainee was 1:7. Once the trainee performed required standard view on the manikins, simulated 3-D images were presented to the trainee to demonstrate various pathological findings under that particular standard view. We also provided standard patients with no pathological conditions to trainees alongside with the simulated manikins. Subjects had the last lecture on the interpretation of pathological ultrasound images with the real clinical case.

The final step of the new curriculum was to review and practice in groups all critical ultrasound diagnostic/treatment procedures (FAST, thoracic, vascular, UGP, and echocardiography) with emphasis on the THIRD protocol for undifferentiated shocks. Instructors presented simulated case scenarios and corresponded ultrasound findings while trainees were obtaining images on models.

### Measurements and outcomes

We conducted a baseline survey of study participants before training for basic demographics and prior ultrasound experiences, which include seven single choice questions and one open-ended question. The participants were requested to rate for the self-confidence in performing of five core applications: FAST, thoracic, vascular, UGP, echocardiography and diagnosing undifferentiated shock (RUSH for old curriculum or THIRD for new curriculum). The confidence measurements ranged from 1 to 5 with 1 being the least confident and 5 being the most confident.

Multiple course instructors assessed trainees’ performance in carrying out the ED ultrasound procedures (FAST, thoracic, echocardiography, and UGP) after training. To give a fair assessment for trainees enrolled in the study, we assigned course instructors to evaluate the performance of trainees who they were not in direct coaching with during hands-on and group sessions. However, we could not completely blind the instructors of the trainees’ seniority and experiences. Instead, we specifically required an objective assessment of the trainees’ skill level using an anchored rating scale as the assessment tool: We assign score of one to participants who were unable to obtain a view and unable to explain normal image even with extensive instructor’s coaching; three when they were able to obtain a low-quality view and give some of the image interpretation with some coaching; five when they were able to gain a standard view with high image quality and make accurate image interpretation without assistance. The total performance score summing up the 4 subcomponents, 36 items ranges from 36 to 180. All course instructors received prior standardized training in the right using of the assessment tool.

One month after the completion of the training, we contacted trainees about the reassessment of self-confidence and post-training ultrasound practices. We chose to follow-up on trainees’ self-confidence 1 month after the training because we believe by that time trainees would have had enough time to digest the learning in the course and make enough attempts to practice POCUS independently.

This survey was administered online using the research electronic data capture (REDCap) medical data system.

### Statistical analysis

An independent statistician analyzed the data. The primary outcome was trainees’ performance measured after the training. Secondary outcomes included self-confidence measured at the completion of training and 1 month after. To control for the differential distribution of baseline self-confidence between groups, we calculated the differences between the post-training and the baseline self-confidence measurements. To compare the traditional curriculum and new goal-directed curriculum, we tabulated trainees’ baseline characteristics and outcomes by training groups, with means and standard deviations presented for continuous variables, and counts and percentages for categorical variables. We carried out univariate statistical comparisons between training groups using the two-sample *t* test supplemented by the two-sample Wilcoxon rank sum test (given the small sample size and skewed distributions of variables) for continuous variables, and the Fisher's exact test for categorical variables.

We also performed multivariate linear regression analyses to examine the independent association between training programs and outcomes while controlling for the potential baseline confounders. Only imbalanced baseline characteristics between the two trainee cohorts were included in the multivariate regression model (either *p* value < 0.05 or relative differences greater than 10%). The statistical significance threshold was set at *p < *0.05. Individuals with missing data were excluded from analysis. All data analyses were performed using STATA 12.0 (StataCorp. 2011. Stata Statistical Software: Release 12. College Station, TX: StataCorp LP.).

## Patient and public involvement

Our study participants were not considered as patients, and there was no other public involvement other than the trainees enrolled in PUMCH emergency POCUS training course. The study’s results were disseminated to the participants who chose to receive updates after.

## Results

### Overall sample

During the two consecutive training programs, we enrolled and consented 66 trainees from over 20 hospitals throughout China. We excluded two trainees who did not finish the course for personal reasons. Twenty-seven trainees enrolled before 2017 received traditional POCUS training, while 37 trainees received training using the new curriculum.

On average, trainees had 5.58 years of practice experiences in ED, and 51.6% had some prior ED ultrasound training. Many indicated that they had performed ED ultrasound before, with the most frequently performed being ultrasound-guided puncture (45.3%), followed by vascular (37.5%), echocardiography (28.1%), and the least performed being diagnostic procedures for undifferentiated shock (7.8%). Similarly, the prior experiences with different ultrasound procedures were also reflected in the corresponding baseline self-confidence, with diagnosing undifferentiated shock being the one with the lowest confidence. However, a small proportion of them indicated that they were independently performing ED ultrasound before training (9.4%) (Table 1).

After training, all trainees reported significantly increased self-confidence in performing ED ultrasound as compared with baseline. One month after training completion, the confidence tended to decrease moderately from the end of training but was still higher than the baseline measurements. For example, confidences in performing shock protocol were 1.50, 3.44 and 2.59 for baseline, training completion and 1 month after training, respectively (Tables 1, 2).

### Trainees’ baseline characteristics by training curriculums

Trainees received traditional/new training programs that differ in several baseline characteristics. Trainees with new curriculum tended to have more years practicing in ED (7.41 vs. 3.07, *p < *0.001), more likely to have prior ED ultrasound training (64.9% vs. 33.3%, *p < *0.001), performed slightly more ED ultrasound before (total amount: 17.35 vs. 13.70, *p = *0.208; counts of types of ED ultrasound: 2.03 vs. 1.19, *p = *0.060). Trainees who received the new curriculum also had higher baseline self-confidence measurements than those who received traditional curriculum (Table 1). However, trainees with new curriculum are less likely to indicate they were independently performing ED ultrasound (8.1% vs. 11.1%) with an insignificant *p* value of 0.691 at the threshold of 0.05.

**Table 1:** Baseline characteristics of trainees before ER ultrasound training programs

Baseline characteristics	All	Traditional program	Goal-directed program	*p* value	*p* value*
Years practicing in ER	5.58 ± 4.56	3.07 ± 2.22	7.41 ± 4.97	0.000	0.000
Prior ER US training (yes)	33 (51.6)	9 (33.3)	24 (64.9)	0.022	
Independently Performing ER ultrasound (yes)	6 (9.4)	3 (11.1)	3 (8.1)	0.691	
Number of ER US performed	15.81 ± 27.75	13.70 ± 23.06	17.35 ± 30.95	0.208	0.591
Number of ER US performed (categorical)					
0–10	45 (70.3)	19 (70.4)	26 (70.3)	0.999	
11–120	19 (29.7)	8 (29.6)	11 (29.7)		
Number of ER US performed per month	2.83 ± 5.44	1.33 ± 2.09	3.92 ± 6.77	0.091	0.034
Counts of types of ER US performed	1.67 ± 2.02	1.19 ± 1.80	2.03 ± 2.13	0.060	0.092
Types of ER US performed					
FAST (yes)	14 (21.9)	7 (25.9)	7 (18.9)	0.551	
Thoracic (yes)	13 (20.3)	2 (7.4)	11 (29.7)	0.033	
Vascular (yes)	24 (37.5)	9 (33.3)	15 (40.5)	0.609	
USP (yes)	29 (45.3)	9 (33.3)	20 (54.1)	0.130	
Echocardiography (yes)	18 (28.1)	4 (14.8)	14 (37.8)	0.053	
Shock (yes)	5 (7.8)	0 (0.0)	5 (13.5)	0.068	
Months since last ER US					
≦6 months	48 (75.0)	21 (77.8)	27 (73.0)	0.682	
> 6 months	14 (21.9)	6 (22.2)	8 (21.6)		
Never performed	2 (3.1)	0 (0.0)	2 (5.4)		
Pre-training self-confidence performing					
FAST	1.77 ± 0.96	1.59 ± 0.84	1.89 ± 1.02	0.210	0.205
Thoracic	1.83 ± 1.05	1.48 ± 0.75	2.08 ± 1.16	0.024	0.015
Vascular	2.03 ± 0.96	1.52 ± 0.75	2.41 ± 0.93	0.000	0.000
USP	2.25 ± 1.18	1.70 ± 0.99	2.65 ± 1.16	0.001	0.001
Echocardiography	1.78 ± 0.90	1.52 ± 0.80	1.97 ± 0.93	0.032	0.040
Shock	1.50 ± 0.76	1.33 ± 0.55	1.62 ± 0.86	0.199	0.109

*Supplemental *p* values calculated from two-sample Wilcoxon rank sum test

### Trainees’ post-training measurements by training curriculums

Trainees with new curriculum had statistically significantly higher measurements in self-confidence after training, reflected in numbers both at training completion and at 1 month after training. Even after adjusting for the differential distribution of baseline self-confidence by calculating the absolute delta of post-training confidence from baseline, the new curriculum was associated with higher trainee self-confidence (post-training vs. baseline and 1-month post-training vs. baseline). For example, in FAST performance, the confidence measurements were 3.92 (new) vs. 3.37 (traditional) for post-training, 3.27 vs. 2.26 for 1-month post-training, 2.03 vs. 1.78 for increased confidence post-training as relative to baseline, and 1.38 vs. 0.67 for increased confidence 1-month post-training as relative to baseline.

Trainees with new curriculum also ended up performing more ultrasound post-training than trainees received traditional curriculum (14.2 vs. 1.44, *p < *0.001) and had higher performance scores at training completion (165.76 vs. 113.44, *p < *0.001).

When adjusting for potential confounding factors such as imbalanced baseline characteristics and baseline confidence levels, the new curriculum was consistently associated with higher increases in self-confidence measured at either training completion or 1 month after (except for ultrasound-guided punctures), more substantial number of ultrasound performed in 1-month post-training, and higher performance scores at training completion (Table 3).

**Table 2:** Outcome assessments after ER ultrasound training

Outcomes	All	Traditional program	Goal-directed program	*p* value	*p* value*
Post-training self-confidence					
FAST	3.69 ± 0.50	3.37 ± 0.49	3.92 ± 0.36	0.000	0.000
Thoracic	3.63 ± 0.65	3.19 ± 0.62	3.95 ± 0.47	0.000	0.000
Vascular	3.69 ± 0.66	3.19 ± 0.56	4.05 ± 0.47	0.000	0.000
UGP	3.72 ± 0.81	3.44 ± 0.58	3.92 ± 0.89	0.002	0.012
Echocardiography	3.58 ± 0.64	3.19 ± 0.62	3.86 ± 0.48	0.000	0.000
Shock	3.44 ± 0.75	2.89 ± 0.64	3.84 ± 0.55	0.000	0.000
One-month post-training self-confidence					
FAST	2.84 ± 0.88	2.26 ± 0.45	3.27 ± 0.87	0.000	0.000
Thoracic	3.16 ± 0.95	2.52 ± 0.58	3.62 ± 0.89	0.000	0.000
Vascular	3.02 ± 1.03	2.22 ± 0.58	3.59 ± 0.90	0.000	0.000
UGP	2.94 ± 1.01	2.30 ± 0.67	3.41 ± 0.96	0.000	0.000
Echocardiography	2.75 ± 1.01	1.89 ± 0.64	3.38 ± 0.72	0.000	0.000
Shock	2.59 ± 0.92	1.85 ± 0.60	3.14 ± 0.71	0.000	0.000
Delta of self-confidence (post-training vs. baseline)					
FAST	1.92 ± 0.82	1.78 ± 0.64	2.03 ± 0.93	0.120	0.208
Thoracic	1.80 ± 0.80	1.70 ± 0.54	1.86 ± 0.95	0.176	0.393
Vascular	1.66 ± 0.74	1.67 ± 0.68	1.65 ± 0.79	0.730	0.922
UGP	1.47 ± 0.84	1.74 ± 0.71	1.27 ± 0.87	0.022	0.021
Echocardiography	1.80 ± 0.74	1.67 ± 0.55	1.89 ± 0.84	0.180	0.203
Shock	1.94 ± 0.81	1.56 ± 0.58	2.22 ± 0.85	0.001	0.000
Delta of self-confidence (1-month post-training vs. baseline)					
FAST	1.08 ± 1.31	0.67 ± 1.00	1.38 ± 1.44	0.004	0.023
Thoracic	1.33 ± 1.38	1.04 ± 0.98	1.54 ± 1.59	0.021	0.124
Vascular	0.98 ± 1.09	0.70 ± 0.87	1.19 ± 1.20	0.027	0.065
UGP	0.69 ± 1.14	0.59 ± 1.05	0.76 ± 1.21	0.526	0.564
Echocardiography	0.97 ± 1.22	0.37 ± 1.08	1.41 ± 1.14	0.000	0.000
Shock	1.09 ± 1.06	0.52 ± 0.89	1.51 ± 0.99	0.000	0.000
Number of ultrasound performed within 1-month post-training					
Total ultrasound	8.83 ± 11.49	1.44 ± 2.04	14.22 ± 12.54	0.000	0.000
FAST	0.97 ± 1.53	0.07 ± 0.27	1.62 ± 1.74	0.000	0.000
Thoracic	2.30 ± 3.93	0.44 ± 0.75	3.65 ± 4.71	0.000	0.000
Vascular	1.91 ± 2.65	0.15 ± 0.60	3.19 ± 2.84	0.000	0.000
UGP	1.00 ± 1.75	0.48 ± 0.75	1.38 ± 2.15	0.279	0.023
Echocardiography	2.89 ± 4.22	0.33 ± 0.78	4.76 ± 4.71	0.000	0.000
Shock	1.00 ± 1.94	0.00 ± 0.00	1.73 ± 2.31	0.000	0.000
Performance score post-training					
Total score	143.69 ± 28.07	113.44 ± 12.80	165.76 ± 8.58	0.000	0.000
FAST	44.84 ± 9.41	35.30 ± 5.78	51.81 ± 3.53	0.000	0.000
Thoracic	28.98 ± 5.24	23.89 ± 2.72	32.70 ± 3.00	0.000	0.000
Vascular	31.20 ± 6.94	24.44 ± 4.20	36.14 ± 3.52	0.000	0.000
Echocardiography	34.58 ± 7.40	26.52 ± 2.24	40.46 ± 2.83	0.000	0.000

*Supplemental *p* values calculated from two-sample Wilcoxon rank sum test

**Table 3:** Differences in outcomes between traditional and goal-directed programs, estimated in multivariate regression model

Outcome	Covariates adjusted	Effect estimate and 95% CI (new goal-directed vs. traditional program)	*p* value
US performance post-training			
Echocardiography performance	Years in ER; prior ER US training; number of ER US performed; number of different ER US performed; baseline confidence of performing echocardiography US; ever performed echocardiography US	14.0 (12.5–15.5)	< 0.001
FAST performance	Years in ER; prior ER US training; number of ER US performed; number of different ER US performed; baseline confidence of performing FAST US; ever performed FAST US	16.2 (13.5–19.0)	< 0.001
Vascular performance	Years in ER; prior ER US training; number of ER US performed; number of different ER US performed; baseline confidence of performing vascular US; ever performed vascular US	11.3 (8.9–13.7)	< 0.001
Thoracic performance	Years in ER; prior ER US training; number of ER US performed; number of different ER US performed; baseline confidence of performing thoracic US; ever performed thoracic US	8.6 (6.9–10.2)	< 0.001
Total performance	Years in ER; prior ER US training; number of ER US performed; number of different ER US performed; baseline confidence (sum of confidence scores before training: FAST, thoracic, vascular, USP, echocardiography, shock)	49.5 (43.3–55.3)	< 0.001
Delta of confidence post-training vs. baseline			
FAST	Years in ER; prior ER US training; number of ER US performed; number of different ER US performed; baseline confidence of performing FAST US; ever performed FAST US	0.4 (0.1–0.6)	0.002
Thoracic	Years in ER; prior ER US training; number of ER US performed; number of different ER US performed; baseline confidence of performing thoracic US; ever performed thoracic US	0.6 (0.3–0.8)	< 0.001
Vascular	Years in ER; prior ER US training; number of ER US performed; number of different ER US performed; baseline confidence of performing vascular US; ever performed vascular US	0.6 (0.3–0.8)	< 0.001
UGP	Years in ER; prior ER US training; number of ER US performed; number of different ER US performed; baseline confidence of performing USP US; ever performed USP US	–0.04 (–0.42 to 0.34)	0.850
Echocardiography	Years in ER; prior ER US training; number of ER US performed; number of different ER US performed; baseline confidence of performing echocardiography US; ever performed echocardiography US	0.4 (0.1–0.6)	0.010
Shock	Years in ER; prior ER US training; number of ER US performed; number of different ER US performed; baseline confidence of performing shock US; ever performed shock US	0.8 (0.5–1.1)	< 0.001
Delta of confidence 1-month post-training vs. baseline			
FAST	Years in ER; prior ER US training; number of ER US performed; number of different ER US performed; baseline confidence of performing FAST US; ever performed FAST US	1.0 (0.5–1.4)	< 0.001
Thoracic	Years in ER; prior ER US training; number of ER US performed; number of different ER US performed; baseline confidence of performing thoracic US; ever performed thoracic US	1.3 (0.9–1.8)	< 0.001
Vascular	Years in ER; prior ER US training; number of ER US performed; number of different ER US performed; baseline confidence of performing vascular US; ever performed vascular US	1.1 (0.6–1.6)	< 0.001
UGP	Years in ER; Prior ER US training; number of ER US performed; number of different ER US performed; baseline confidence of performing USP US; ever performed USP US	0.7 (0.2–1.3)	0.009
Echocardiography	Years in ER; prior ER US training; number of ER US performed; number of different ER US performed; baseline confidence of performing echocardiography US; ever performed echocardiography US	1.4 (1.0–1.9)	< 0.001
Shock	Years in ER; prior ER US training; number of ER US performed; number of different ER US performed; baseline confidence of performing shock US; ever performed shock US	1.3 (0.8–1.7)	< 0.001
Number of ER US performed within 1 month after training	Years in ER; prior ER US training; number of ER US performed per month before training; number of different ER US performed; baseline confidence (sum of confidence scores before training: FAST, thoracic, vascular, USP, echocardiography, shock)	11.7 (5.6–17.8)	< 0.001

## Discussion

Bedside ultrasound has become a vital tool to aid diagnosis and treatment in the ED. Better utilization of bedside ultrasound has also been associated with improved patient outcomes and decreased costs in the ED [2, 16–20]. However, multiple studies have recognized a gap of POCUS utilization in ED and identified the lack of adequate training as the primary barrier for effective usage of POCUS [21, 22]. In a 2014 national survey of US critical care fellowship directors, the authors indicated even in a country like the United States, which have developed guidelines for POCUS decades ago, there were significant variations of the existing curriculums and a dire need for an improved curriculum to close the gap in POCUS training [4]. In developing countries in which resources are scarce, ED physicians face more challenges in adopting POCUS in their daily practices.

First, our study quantitatively assessed the gap of POCUS expertise in Chinese ED physicians. Although many trainees had prior experiences performing POCUS before the training, a tiny proportion of them indicated that they were independently performing ultrasound examinations, and on average, the self-confidence levels were all lower or close to a rating of two (“no confidence but willing to try”) before training. When it comes to complex bedside ultrasound procedures such as RUSH or THIRD, It is not surprising that only 7.8% of trainees had ever performed POCUS to aid diagnosis of undifferentiated shock in ED practice. At the time of the study, most Chinese ED programs relied primarily on informal bedside teaching for ED POCUS without a proper ultrasound curriculum in place. The training team is rarely staffed with enough experienced course instructors and uses outdated teaching materials.

Second, this study has demonstrated that a two and a half day ultrasound course using a new curriculum can effectively train ED clinicians in POCUS skill. Compared with the traditional training curriculum, trainees with the new curriculum had higher performance ratings even after adjustment for multiple confounders. The new curriculum was also associated with increased self-confidence at the completion of the training, which persisted when re-evaluated in 1 month after. Interestingly, the treatment effect (the new curriculum vs. the traditional) in the delta of self-confidence was of greater magnitude when measured in 1 month after training, compared with that measured at training completion. We hypothesize that the new curriculum allowed trainees with greater confidence after training completion and encouraged them to perform more POCUS procedures, which snowballed and provided additional positive feedbacks in increasing ones’ self-confidence.

Third, we designed and tailored the new curriculum to meet the greater need for effective and efficient ED POCUS training programs in low- and middle-income countries like China. Compared with North American and European countries, developing countries face more challenges of limited resources and heavy patient workload. Curriculums in developed countries usually mandate a low student to instructor ratio (no higher than 5–1), may require pathology models to include standard patients with pathological conditions, and usually take a longer time to complete (half-day for a single application) [6, 15, 23]. When developing the new curriculum, we have integrated other countries’ experiences of designing a POCUS training program in a limited resource setting [8, 21, 24–26]. We have identified the commonly utilized POCUS procedures in Chinese ED and designed the intense training sessions accordingly. Our curriculum was shorter, more focused on the selective list of procedures, and maintained relatively high students to instructors’ ratios.

We believe that the increase in performance rating, number of POCUS performed after training, and improved self-confidence can be attributable to multiple improvements we made to the curriculum. The main change of the new curriculum was the inclusion of the procedure-focused training for Fast, Thoracic, vascular, USP, echocardiography and undifferentiated shock. Trainees were trained to operate ultrasound on standard manikins and patients, perform the above standard procedures while presented with simulated clinical scenarios. We believe the simulated scenarios are more reflective of a real clinical setting and boost the retention of new skills and information with lower technique requirement on simulating resource [27].

In the new curriculum, we experimented with the concept of the flipped classroom by providing the teaching materials and instructional contents before the didactics. The utilization of flipped classroom provided the opportunity for more time spent in the didactics reviewing and coached practices so that the curriculum could achieve better results within limited training time [23]. Also, we believe inserting hands-on practice before the lecture of pathological images also helped trainees better understand the anatomical structure, so that they could relate the ultrasound maneuvers to abnormal findings on the images obtained. As noted in previous guideline [13, 23, 28], rotating the skill learning and didactics sessions also improves the efficiency of the training program. Lastly, the availabilities of interactive, 3-D images made it possible to present simulated pathological findings while trainees were practicing standard views on manikins, without having real standardized patients available for every pathological condition.

There are several limitations to our study. There was no randomization of trainees’ allocation to the old or new curriculum, which resulted in the differential distribution of baseline characteristics of trainees. Especially for trainees’ performance measurement, we were not able to collect the baseline measurement to be controlled for. However, the treatment effect of our intervention on outcomes remained robust when evaluated as a delta from baseline, or in multivariate regression models adjusted for the imbalanced baseline characteristics. We acknowledge the potential existence of residual confounding. Its relative small sample size also limits this study, and the training curriculum was only developed and evaluated in a single institution. However, our recruitment of trainees was open to hospitals nationwide, and our results confirmed a broad distribution of physicians with different seniority and experiences of POCUS. We expect our findings to generalize well in other institutions.

In our study, we have not directly measured the curriculums on all the Kirkpatrick’s four level evaluation matrices. We have focused more on the behaviors and results aspects by measuring the changes in self-confidence performing POCUS, and number of cases performed after the training. Future researches are going to provide more detailed information revealing the subjective reactions of the trainees, and the reasons why they have/have not achieved the learning goal. Those additional measurements will provide further insight to find the root cause of success and failures in teaching POCUS at the bedside in ED, and help improve the new curriculum.

## Conclusion

This study suggests that a goal-directed, intensive but brief (2.5 day) ED POCUS curriculum is associated with a significant increase in trainee self-confidence and performance. The focused training on critical POCUS procedures commonly used in the ED setting has contributed to improved ED POCUS skills. Further study is needed to identify the optimal allocation of time between lectures and hands-on practices and explore other enhancements for the curriculum.

## Abbreviations

US: Ultrasound
POCUS: Point-of-care ultrasound
ED: Emergency department
EM: Emergency medicine
ER: Emergency room
PUMCH: Peking Union Medical College Hospital
RUSH: Rapid ultrasound for shock and hypotension
FAST: Focused assessment with sonography in trauma
UGP: Ultrasound-guided puncture
THIRD: Tamponade, heart, inferior vena cava, respiratory system, deep venous thrombosis
REDCap: Research electronic data capture
